# A feasibility study of gamma ray source finder development for multiple sources scenario based on a Monte Carlo simulation

**DOI:** 10.1038/s41598-021-85706-4

**Published:** 2021-03-17

**Authors:** Ali Farzanehpoor Alwars, Faezeh Rahmani

**Affiliations:** Department of Physics, K. N. Toosi University of Technology, P.O. Box 15875-4416, Tehran, Iran

**Keywords:** Engineering, Physics

## Abstract

In this research, a rapid and accurate gamma finder system which can find orphan gamma-ray sources has been designed based on NaI(Tl) detector. By proposing complementary methods to our previous researches, an attempt has been made to provide an approach to solve the problem of 3D localization in multiple orphan gamma-ray sources scenario. Based on our previous research, the new conceptual design has been proposed and simulated using Monte Carlo MCNPX2.6 code. Afterward, in order to identify its key characteristics and features, the proposed design has been tested in several different scenarios (multiple gamma sources with various activities in different distances).

## Introduction

In the nuclear industry and related applications, a problem of misplaced or lost gamma-ray sources is considered as a cardinal concern. A lost gamma-ray source, depending on its energy and activity, can be a severe danger for technicians as well as the public environment.

Until now, several accidents and injuries due to orphan (lost) gamma-ray sources have been reported in Johnstone’s archive^1^. Goiania 1987^2^, Gilan 1996^3^, Ecuador 2009^4^ are some examples of orphan gamma-ray source accidents. Albeit in most cases, there is only one gamma-ray source was lost in the environment, but it is not inevitable that multiple gamma-ray sources (similar or different in energy, activity, and position) will be lost in the environment, which is called multiple orphan gamma-ray sources scenario.

In an accident of orphan gamma-ray source, the designed gamma finder system should work in various conditions without any fatal error (also with an acceptable precision). As mentioned above, one of this special conditions are getting lost of one or several gamma-ray sources in radiation environment, such as a laboratory, or hospital, in which other gamma-ray sources are working. So in this case, the system must be able to find the gamma-ray orphan source in the presence of high background of gamma-ray (due to the other gamma-ray sources in the laboratory). Another condition is the presence of multiple gamma-ray sources. In other words, an ideal and perfect system should be designed in such a way that all aspects and conditions of accidents are considered with related solutions.

Developing an instrument to exert precise, fast, and user-friendly method to find multiple orphan gamma-ray sources can help to overcome the mentioned consequences and risks. Until now, several methods and instruments in order to find orphan gamma-ray sources have been proposed and designed^5–15^. Some of these designed systems are based on a gamma-ray detector (usually a scintillation detector) and a collimator (high-Z material, e.g., tungsten (W), lead (Pb), and so on) ^9,11,13^. From the most serious constraints of such systems are their high weight, low speed and low coverage of solid-angle. In addition to these, such designed system can only locate one orphan γ -ray source at a time. There are other different designs for gamma finders, which usually based on two, three or more scintillation detectors, which equipped with special and unique methods and until now, they have reached their highest level of development approximately^5–8,10,12,14,15^. Since gamma-ray scintillation detectors are so common, and most of them are affordable, the employed gamma-ray detector is mostly a scintillation detector such as NaI(Tl), CsI(Tl), BGO, LaBr_3_(Ce) and so on. But still, the proposed methods cannot find multiple orphan gamma-ray sources in 3D and each of them has its own restrictions. Conventional proposed methods in instruments are mostly based on total recorded gamma-ray counts^7–15^. Therefore, they cannot be able to deploy in the multiple orphan gamma-ray sources scenario. A detailed review of them can be found in^16^.

In our previous published works^16,17^, an instrument has been proposed with unique methods to find a single orphan gamma-ray source. The proposed methods were based on photopeak net count of gamma-rays. In this research, extended new method based on the previously proposed method^16,17^ is presented, which can be an efficient and capable in multiple orphan gamma-ray sources scenario. It is worth noting that the purpose of this research is to find orphan gamma-ray sources in a finite space (i.e., a 20 × 20 × 6 m room) which can indicate a laboratory, a storeroom, or any related environment. Based on the reports^5–15^, none of the reported current systems are able to work properly in multiple orphan gamma-ray sources scenario, or their precision is too low to be considered as a useful instrument in these accidents. Therefore, in this research some attempts have been made to develop the previous algorithms and the system, in order to be useful and applicable in the multiple gamma-ray sources scenarios.

## Materials and methods

After the prosperous design of gamma finder in the last published works^16,17^, it was interested in completing it in a multiple sources scenario. The designed system would be able to identify orphan gamma-ray sources, determine the angular positions and spatial positions (in 3D space), as well as the activity of sources. In the mentioned works, the performance of the system was examined in the presence of ^137^Cs and ^241^Am gamma-ray point sources, separately. Here, by introducing some idea to the previously proposed methods, precision, and accuracy of the system in the presence of multiple (two, three, or more) gamma-ray sources will be investigated and several cases will be studied. The main improvements of the gamma finder system in this research is the development of software and finding methods. This was done by studying the performance of the gamma finder system (precision & accuracy) in presence of high gamma-ray radiation background, which is due to the presence of the other gamma-ray sources in the environment.

The considered sources are presented in Table 1^18^. The most common gamma-ray sources in nuclear engineering and radiation application have been selected. Therefore, the aim of the extended methods is to find multiple orphan gamma-ray sources, which is mostly being lost in a limited space such as hospitals or laboratories with energy ranges from ~ 0.050 MeV to ~ 3 MeV.

**Table 1:** Characteristics of common radioisotopic gamma-ray sources^18^.

Radioisotope	Energy of emitted gamma-rays (MeV)	Branching ratio (%)	Half-life (year)
^57^Co	0.122	87	0.739
^203^Hg	0.279	77	0.128
^113^Sn	0.393	64	0.315
^103^Ru	0.497	88	0.109
^133^I	0.530	90	0.002
^137^Cs	0.662	85	30.0
^95^Nb	0.765	100	≈ 0.095
^54^Mn	0.834	100	0.830
^60^Co	1.173 & 1.332	100 & 100	5.260
^40^ K	1.460	11	1.290 × 10^11^
^24^Na	1.369 & 2.754	100 & 100	≈ 0.001

The system consists of a Ø2″ × 2″ cylindrical NaI(Tl) scintillation detector (crystal size), two fake Ø2″ × 2″ detectors, a small, robust drone (to elevate, horizontal (XOY) rotate and move the system). These two fake detectors are the same size as the NaI(Tl) scintillation detector with NaCl instead of NaI(Tl) crystal^16^. The total mass and volume of the gamma finder system including two fake detectors, NaI(Tl) detector, polyethylene container, electronic components and equipment is less than 14 kg and 8500 cm^3^, respectively.

**Figure 1:**
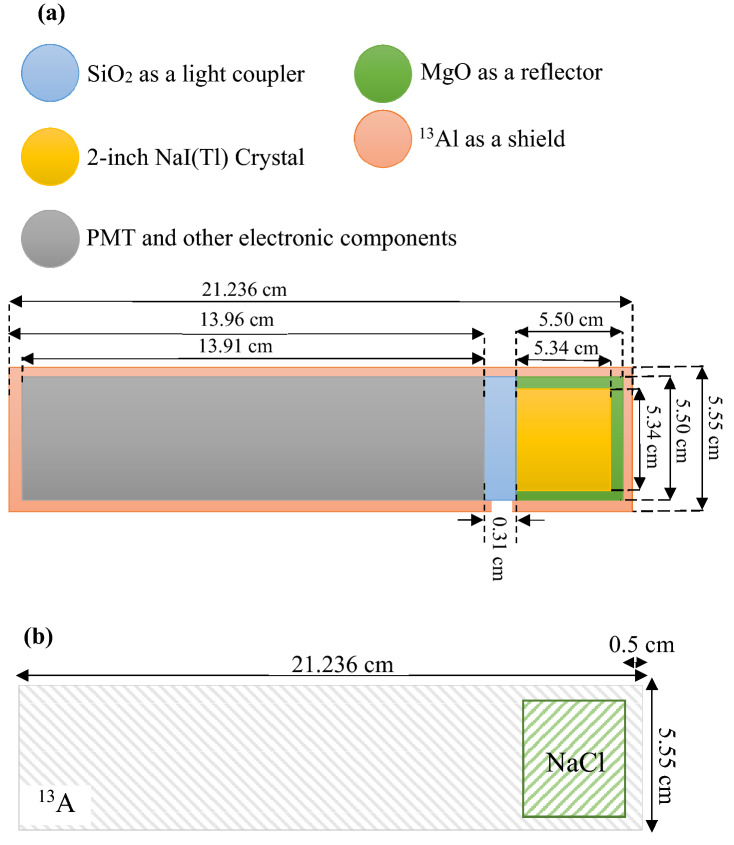
(**a**) Schematic view of the simulated Ø2″ × 2″ NaI(Tl) scintillation detector. (**b**) Schematic view of the simulated Ø2″ × 2″ fake detector.

**Figure 2:**
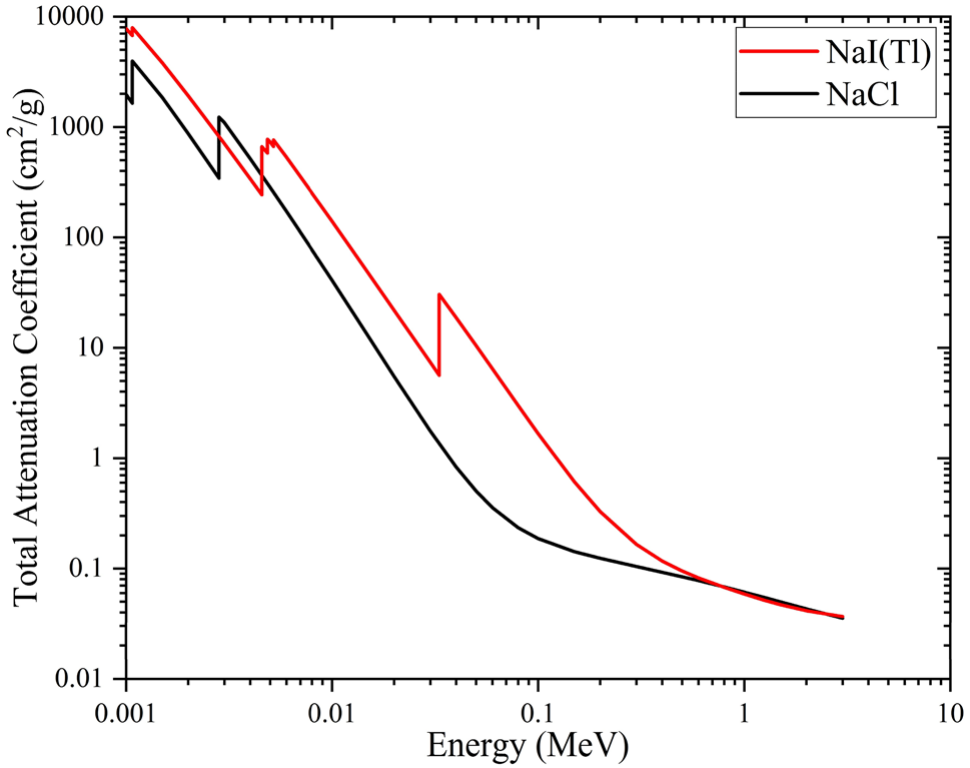
Total attenuation coefficient (*μ*/*ρ*) Vs. energy of incident gamma-rays for NaI(Tl) and NaCl.

The real cylindrical NaI(Tl) scintillation detector system (scintillator crystal, Photo Multiplier Tube (PMT), SiO2 (as a light coupling between PMT and scintillator crystal), MgO (reflector), and related electronics components such as HV and preamplifier) has a length of 21.2 cm and a diameter of 5.5 cm. The considered NaI(Tl) detector can distinguish the reported gamma-ray sources in Table 1 simultaneously (with considering FWHM of the detector). Each of the fake detectors with similar appearance to the real NaI(Tl) detector is filled with Al, and a NaCl instead of NaI(Tl) crystal (a Ø2″ × 2″ cylinder). The schematic views of the real NaI(Tl) scintillation detector and the fake detector are illustrated in Fig. 1. However, there is a little difference between interaction of gamma-rays with NaI(Tl) and NaCl in simulations (Fig. 2), which can be ignored. Also, NaCl can be a suitable candidate to play the role of shadowing effects instead of expensive NaI(Tl) crystals. According to the above mentioned description and in order to use the previously obtained reference plot (data bank), the use of the fake detectors seems to be necessary as well as efficient.

**Figure 3:**
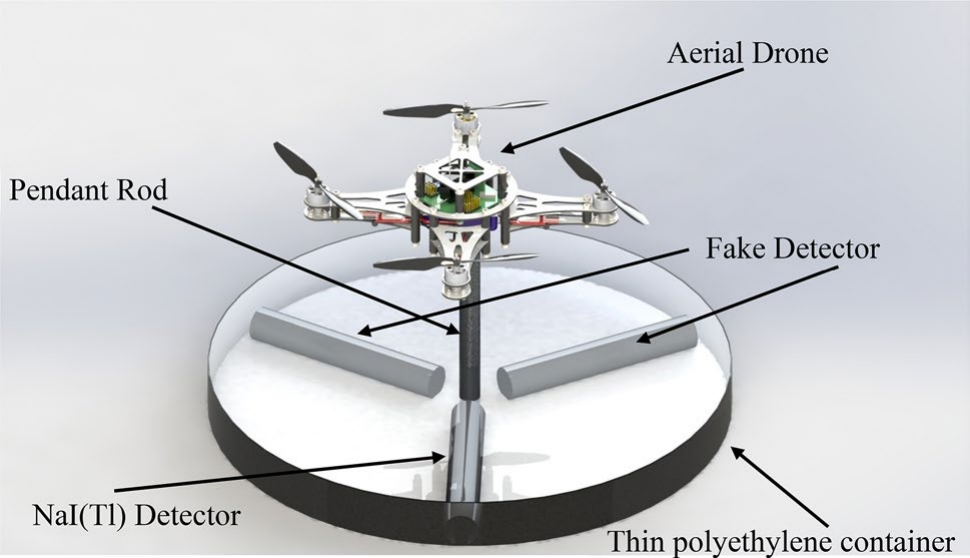
SolidWorks rendered graphical illustration of the conceptual design of gamma finder system (drawings is not to the scale).

The 3D-rendered model of the system designed by SolidWorks 2014^19^ is shown in Fig. 3. The considered drone is a concept, and its hardware properties (battery, rotor type, materials, and so on) will be determined and selected in details in the future. These two fake detectors implement the shadowing effects of the real body of NaI(Tl) detectors^16^. On the other hand, the use of these two fake detectors instead of real NaI(Tl) detector leads to decrease the complexity of the system in data acquisition as well as lowering the final cost of the system, because they do not need any cable connection, data acquisition system, high-voltage and so on. Detailed information about these two implemented fake detectors can be found in our previous research^16^.

**Figure 4:**
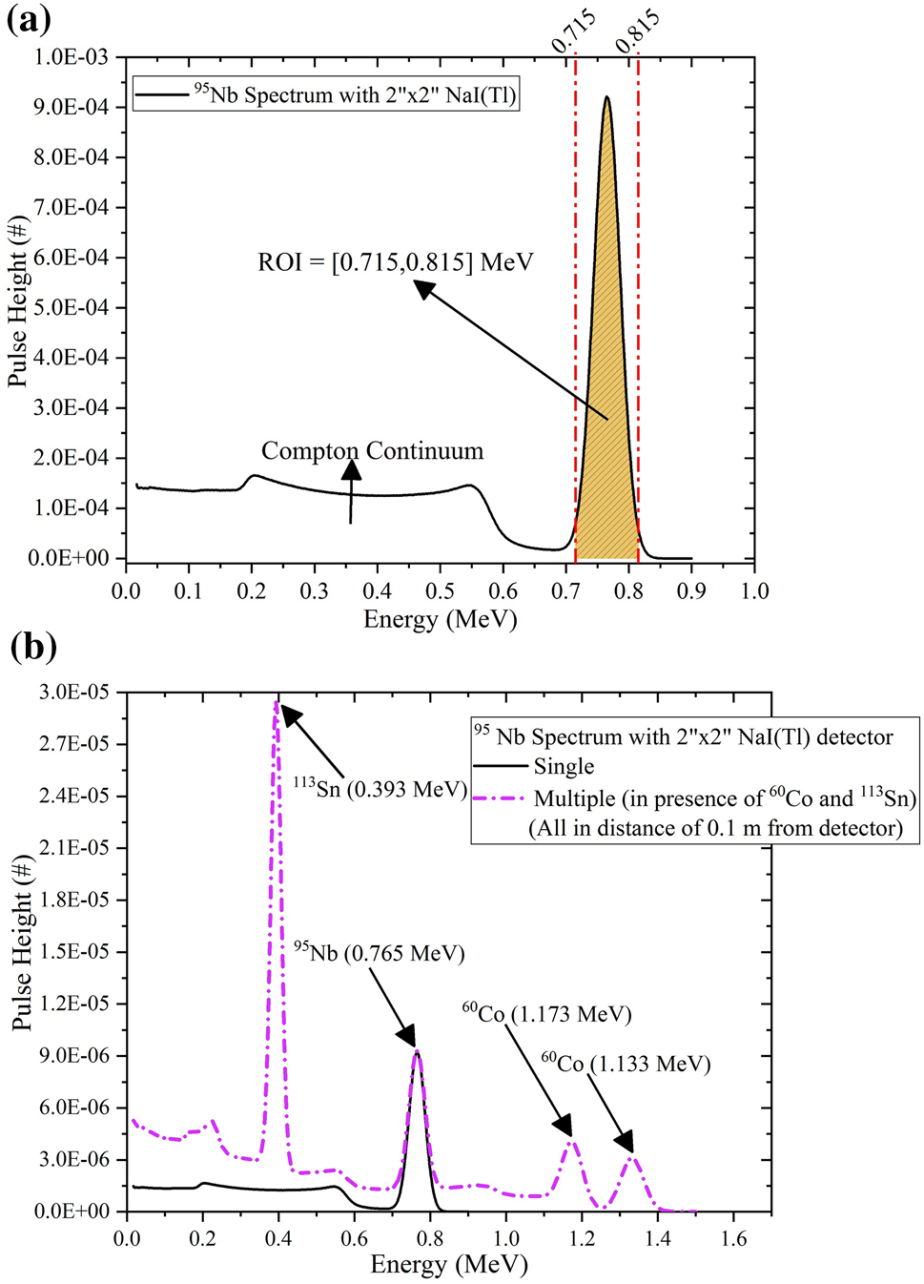
(**a**) MCNPX-simulated spectrum (per particle) of a single isotropic ^95^Nb gamma-ray source in distance of 1 cm of Ø2″ × 2″ cylindrical NaI(Tl) scintillation detector. (**b**) MCNPX-simulated spectrum (per particle) of an isotropic ^95^Nb gamma-ray source in presence of a ^113^Sn and a ^60^Co (multiple sources scenario), all in distance of 10 cm of Ø2″ × 2″ cylindrical NaI(Tl) scintillation detector.

The procedure of identifying the type of gamma-ray source, finding the angular position, the spatial position, and the activity of orphan gamma-ray source is similar to the last published works^16,17^. A typically recorded gamma-ray spectrum of a single ^95^Nb (isotropic in the distance of 0.01 m from the detector) using the MCNPX-simulated^20^ Ø2″ × 2″ NaI(Tl) scintillation detector and its ROI is shown in Fig. 4a. The whole calculations in the proposed methods are based on full energy photopeaks (summation of net counts in ROI), therefore it can be used for gamma-ray sources with wide range of energies. Also, another spectrum of a ^95^Nb in presence of ^60^Co and ^113^Sn, all in distance of 0.1 m from the NaI(Tl) detector is shown in Fig. 4b.

In each of the above simulations, the spectrum of gamma-rays in scintillation crystal is obtained using F8 tally (the specific estimator for pulse height determination of radiation detector in MCNPX) based on energy card (states as Multi-Channel Analyzer in the spectroscopy detector) and employing the Gaussian Energy Broadening (GEB) card in NaI(Tl) cell. The role of the GEB card in MCNPX simulations is the broadening the response of detector based on the statistical phenomena of radiation in the physical detector. Further information about the GEB card that used in this study can be found in Farzanehpoor Alwars et al.^16^.

### Identification of type of orphan gamma-ray sources

The implemented NaI(Tl) detector is assumed to be energy-calibrated using ^57^Co, ^137^Cs, ^60^Co, and ^24^Na. Therefore, each channel of detector represents a registered energy. In other word, there is a one-by-one correspondence between the detector’s channel and registered energy.

The first stage in searching orphan gamma-ray sources is to identify the type of gamma sources, which is done using determination energy of incident gamma-rays. By matching registered gamma-rays energies with existing and available nuclear data libraries, the type of gamma-rays will be identified. It is worth mentioning that, if two, three, or more dominant energies are registered in NaI(Tl) cell, as long as the full energy photopeaks of them can be separated from each other (dependent on detector’s FWHM), the type of gamma-ray sources can be identified in the same way.

Compton continuum in the gamma spectrum due to Compton interaction in NaI(Tl) can be a stubborn troublemaker item in identifying the types of orphan gamma-ray sources. For example, suppose two gamma-ray sources, i.e., a ^113^Sn and a ^54^Mn, are lost in the environment. ^113^Sn places in the Compton continuum arising from ^54^Mn, so the net count of ROI of ^113^Sn is affected according to the Compton continuum from higher energies. Either the ^54^Mn source is too close to detector than ^113^Sn, or its activity is much larger than ^113^Sn, then the net count of ROI of ^113^Sn will be laid under the ^54^Mn Compton continuum. Therefore, the designed system cannot recognize it. In addition, the relative height difference between gamma-ray sources may deteriorate this situation. Therefore, relative activity, distance and the height difference of sources may dictate severe restrictions for the system. Noticed that, for those gamma-ray sources with a short half-life (for example a few minutes to a few hours) e.g., ^133^I and ^24^Na, after identification of them by the gamma finder system, some considerations due to their decay (disintegrations) and their short half-lives must be taken into account in the following calculations. In addition to this, after recognizing the presence of the mentioned gamma-ray sources in the environment (in case of the short half-life), if operational and working condition are allowed, one way is to wait for a certain few minutes or hours (which is dependent on the half-life of the gamma-ray source), and then activity of them will be reduced significantly. But in general, by taking into account the above mentioned considerations, it is possible to find position and activity of them.

### Determination of the angular position of orphan gamma-ray sources

The second stage in finding orphan gamma-ray sources is to determine the angular position of the orphan gamma-ray sources. The angular position is the angle between the central axis of the NaI(Tl) detector and position of the orphan gamma-ray source. Therefore, after identifying types of orphan gamma-ray sources, the system should find the angular positions of them. Net count of an isotropic point source versus orientation of detector, namely angular profile in reference (or laboratory) condition, is achieved. Obviously, the angular profile in practical circumstances may have a phase difference with the angular profile of the reference conditions. Measuring the phase difference can lead to determine the angular position of the orphan gamma-ray source. Detailed information about finding the angular position of a single orphan gamma-ray source can be found in the last published work^16,17^.

So in first stage, a reference plot (angular profile) is obtained for each common source listed in Table 1. For this purpose, for each source in a reasonable distance, i.e., 1.0 m from the detector, the system should rotate about z-axis in XOY plane with small angular steps (user-defined angle such as 1, 2, or 5°), and the net count of ROI of the considered source is recorded for a determined duration, i.e., 180 s in each step. It is evident that if the orphan gamma-ray source is placed in a closer (farther) distance, the amplitude of the obtained plot will have larger (smaller) ^16^. The larger the relative height difference between source and the central axis of the detector, the slope of changes in obtained plots closer to zero. In significant relative height differences, the whole plot will be flat, and therefore, the system cannot be able to determine the angular position of orphan gamma-ray source anymore.

**Figure 5:**
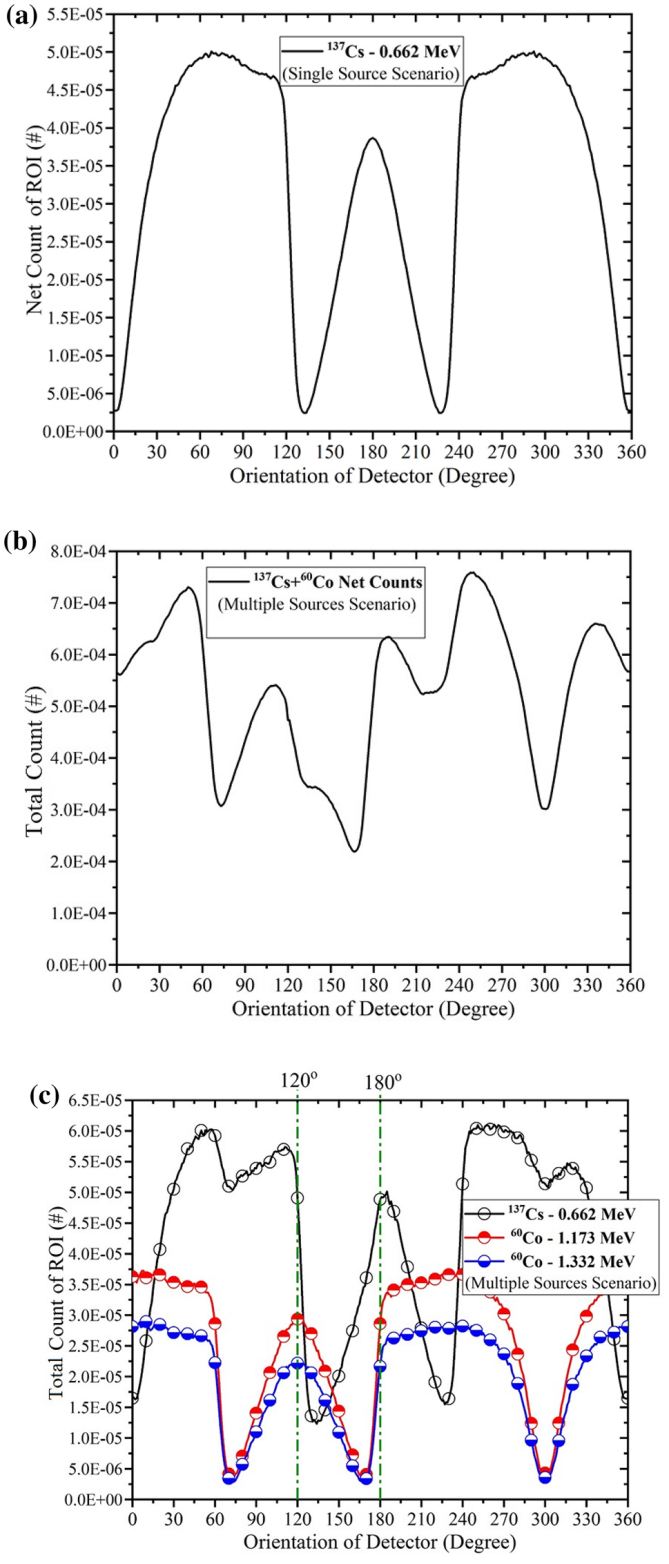
(**a**) net count of ROI (per particle) in presence of a single isotropic ^137^Cs gamma-ray source versus orientation of detector (single source’s scenario). (**b**) total count (per particle) in presence of an isotropic ^137^Cs gamma-ray source and an isotropic ^60^Co gamma-ray source (simultaneously, multiple sources’ scenario). (**c**) total count of ROI of gamma-rays (per particle) which is due to presence of ^137^Cs and ^60^Co versus orientation of detector.

After the preparation and completion of the angular profile, the system is ready to determine the angular position. The system starts rotating about the z-axis in the XOY plane with certain user-defined angular steps. In each step, the net count of ROI is recorded. If more than one type of orphan gamma-ray sources exists in the environment, the net count of ROI of each of them must be recorded. As mentioned before, unavoidable Compton continuum due to higher energy sources will always affect on the net count of ROI of the lower energy source. Typical net count of ROI of single isotropic ^137^Cs source versus orientation of detector (single-source scenario) is shown in Fig. 5a and typical total count of ROI of ^137^Cs and ^60^Co (simultaneously presence in multiple sources’ scenario) is shown in Fig. 5c. It is assumed that ^137^Cs and ^60^Co with equal activities (with considering branching ratio in decay) are located in $\overrightarrow {{r_{1} }} = \left( {100, 0, 0} \right)$ cm and $\overrightarrow {{r_{2} }} = \left( {100\cos \left( {\frac{\pi }{3}} \right), 100\sin \left( {\frac{\pi }{3}} \right), 0} \right)$ cm, respectively. In addition, the related total net count is shown in Fig. 5b.

As mentioned above, the total count of ROI of ^137^Cs is summation of ^137^Cs itself, and part of the Compton continuum of ^60^Co. However, no valuable information about the angular position of orphan gamma-ray sources can be extracted from Fig. 5b, but Fig. 5c can lead to determine the angular position. The phase difference can be measured with Figs. 5a,c with angular profile, which is equal to find the angular position. For example, according to Fig. 5c, there is a phase difference equal to π/3 with reference plot (angular profile), so the angular position of ^60^Co would be 60°. The fundamental of selecting the angular position (i.e., 60° or −60°) is based on the direction of rotation, clockwise or counterclockwise, which here it is considered counterclockwise. Effects of different energies, activities, distances, and height differences of an orphan gamma-ray source (compared to angular profile) in amplitude and behavior of plots have been studied in the last published work^16^.

There is a slight difference between the ^137^Cs plot in Fig. 5a,c in 180° degree. In fact, the peak of ^137^Cs plot in 180° in a single orphan gamma-ray source scenario has been moved in a multiple orphan gamma-ray sources scenario. The reason obviously lies under the presence of ^60^Co in ${ }\overrightarrow {{r_{2} }}$. In the absence of ^60^Co, the total count of ROI of ^137^Cs reaches a local maximum, and then it is decreasing. In the presence of ^60^Co, the Compton continuum of ^60^Co in angles greater than 180° is faced with a semi-global maximum and it does not let the 180° peak of ^137^Cs drop slightly. Therefore, the ^137^Cs peak is expected to shift slightly. In multiple sources scenario, it is recommended that, the phase difference between two local maximum of peaks to be considered.

### Determination of spatial position of orphan gamma-ray sources

The third stage is the determination of the spatial positions (3D localization) of orphan gamma-ray sources. After determining the angular position of each orphan gamma-ray sources, the system can determine the spatial position of each of the sources. Considering the possibility of multiple ROIs, so user should select one of them each time. The central axis of the NaI(Tl) detector will be rotated along the determined the angular position, and the total count of the desired ROI will be recorded (*c*_1_). Then, the system moves horizontally by step *d* along the angular position, and the total count of the desired ROI will be recorded again (*c*_2_). Thereupon, position of orphan gamma-ray source, will be calculated by (1)^10,17^.

1 $$x = \frac{d}{{1 - \sqrt {\frac{{c_{1} }}{{c_{2} }}e^{\mu d} } }}$$

where *μ*_env_ (in cm^-1^) is linear attenuation coefficient of environment (for example, *μ*_air_ = 9.34 × 10^–5^ cm^-1^, for 0.662 MeV gamma-rays), and position of orphan gamma-ray source, *x* (in cm), can be calculated. Generally, *μ*_env_ is energy-dependent, but for the low-density environment (for example, air with density of 0.00125 gr/cm^3^) as well as for energy of gamma-rays higher than 0.05 MeV, *μ*_env_ can be assumed constant. In a multiple sources scenario, an undesired and unavoidable contribution in the calculation of *c*_1_ and *c*_2_ for lower-energy gamma-rays is due to the Compton continuum of higher-energy gamma-rays. To help better understanding it, consider an example; suppose there are two orphan gamma-ray sources in an environment, ^95^Nb (0.765 MeV) and ^113^Sn (whether in a same position or not). The Compton continuum of ^95^Nb (Fig. 4) with edge of about 0.510 MeV expands up to lower energies and it covers the photopeak energy of ^113^Sn (see Table 2 for following coefficients), so C_1m_ will be greater than C_1s_ definitely (C_1m_ > C_1s_) and obviously c_0_ is remained unchanged in a single or multiple (here, double) orphan gamma-ray sources scenario. So in general, the *c*_1_ in Eq. (1) will have an increase related to the single gamma-ray source scenario (C_1m_ > C_1s_). Therefore, the $c_{1} /c_{2}$ ratio in Eq. (1) in a certain multiple gamma-ray sources scenario is greater than the same ratio in a single gamma-ray source scenario. So, the determined spatial position in a multiple orphan gamma-ray sources scenario is smaller than the determined spatial position in a single source scenario. In these explanations, the half-lives of the presented gamma-ray sources in the environment, has been considered long enough to not shown any significant changes during the finding operation.

**Table 2:** The symbols used to explain an example for Eq. (2).

Symbol	Description
C_1s_	The net count of ROI for ^113^Sn in a single source scenario
C_1m_	The total count of ROI for ^113^Sn in multiple (here, double) sources scenario
c_1_	The total count of ROI of the orphan gamma-ray source in distance of $\left| {\vec{x}} \right|$ from detector
c_2_	The total count of ROI of the orphan gamma-ray source in distance of $\left| {\vec{x} - \vec{d}} \right|$ from detector
c_0_	The total (or net) count of ROI for ^95^Nb in multiple (here, double) sources scenario

### Determination of the activity of orphan gamma-ray sources

The fourth and the last stage is the determination of the activity of orphan gamma-ray sources. Activity (in Bq) of each orphan gamma-ray source can be calculated using (2) ^17^.

2 $$A = \frac{N}{{f \cdot T \cdot \varepsilon_{{\left( {\vec{r},E_{0} } \right)}} }}$$

where *N* is net count of ROI due to the orphan gamma-ray source with energy of *E*_0_ in distance of *r* (in cm), which emits *f* gamma per disintegration and *T* (in sec) is live time of gamma-ray spectroscopy in detector.

Note that, Eq. (2) is quietly true and accurate, when *N* is only due to the orphan gamma-ray source itself. In multiple orphan gamma-ray sources scenario, it is an improbable expectation. As mentioned in the last sections, in multiple orphan gamma-ray sources scenario, *N* is not only due to the orphan gamma-ray source itself. It includes a summation of the desired total count of ROI of gamma-ray sources and the Compton continuum of all present orphan gamma-ray sources, which have energies higher than energy of desired orphan gamma-ray source. Therefore, in case of multiple orphan gamma-ray sources, it is suggested to calculate the activity of the orphan gamma-ray source with the highest photopeak energy among all present sources and right after that, seal and exterminate it, and tackling the next remained source with highest energy among the rest.

### Minimum Detectable Activity calculation

The Minimum Detectable Activity (MDA) of a radiation detector (for example, NaI(Tl) scintillation detector), can be calculated by (3)^21^.

3 $${\text{MDA}} = \frac{{4.65\sqrt {N_{B} } + 2.71}}{{f \cdot T \cdot \varepsilon_{{\left( {\vec{r},E_{0} } \right)}} }}$$

where, *N*_B_ is the count of background, *T* is live time of radiation detector (*T*), *f* is branching ratio of the gamma-ray source (for example, for ^137^Cs it would be equal to 0.85) and $\varepsilon_{{\left( {\vec{r}, E_{0} } \right)}}$ is efficiency of the radiation detector for incident gamma-rays with energy *E*_0_ which located in position of $\vec{r}$.

Due to the fact that derivation of Eq. (3) is done by statistical consideration of the Poisson distribution, and MCNPX results are reported per event, so all of the simulation results should be multiplied by number of events based on activity for the use in (3). Here, for instance, the activity of 3.7 × 10^5^ Bq (10 μCi) has been employed for each pair of studied gamma-ray sources. Here, in (3), *N*_B_ increases by time (*T*) and decreases by inverse-squared law ($\left| {\vec{r}} \right|^{ - 2}$); therefore, it is proportional to $T \cdot \left| {\vec{r}} \right|^{ - 2}$. Considering that absolute efficiency also decreases with inverse-squared law ($\left| {\vec{r}} \right|^{ - 2}$), it can be concluded that the MDA in (3) is proportional to $\left| {\vec{r}} \right| \cdot T^{{ - \frac{1}{2}}}$. By giving a fixed (constant) measurement time, the MDA is assumed dependent only on the magnitude of distance ($\left| {\vec{r}} \right|$).

### Derivation of a mathematical expression for the effects of the relative height difference

It is obvious that, in a real accident, all of the orphan gamma-ray sources are not placed in as height as the central axis of the detector. In addition, the relative height difference between the orphan gamma-ray sources is an important factor in determining the angular positions, the spatial positions, and their activities. Albeit the study of this factor is not as straightforward and easy as two previous factors, there is an attempt to show the effectiveness of this factor in the following. In fact, the relative height difference between the orphan gamma-ray source and the central axis of the detector is affected by two factors; (I) increasing distance, (II) decreasing solid-angle. In other word, if the relative height difference between the orphan gamma-ray source and the central axis of the detector becomes greater, then the subtended solid-angle between the orphan gamma-ray source and the detector will decrease, and the magnitude of the distance of the orphan gamma-ray source to the detector will increase. In mathematical terms, (4) is straightforward:

4 $$d_{{\text{h}}} = \sqrt {h^{2} + d^{2} } \to \frac{{d_{{\text{h}}} }}{d} = \sqrt {1 + \left( \frac{h}{d} \right)^{2} }$$

where, *h* is relative height difference between orphan gamma-ray source and the central axis of the detector, *d* is the horizontal distance between the orphan gamma-ray source and the detector and *d*_h_ is the total distance of the orphan gamma-ray source from the detector (by considering the horizontal distance and the relative height difference).

Solid-angle subtended by a point source located along the axis of a right circular cylindrical detector, Ω is given by (5) ^21^.

5 $${\Omega } = 2\pi \left( {1 - \frac{d}{{\sqrt {d^{2} + a^{2} } }}} \right)$$

where, *d* is the source to detector distance, and *a* is the radius of the detector. In the case of d >  > a, Ω can be written as (7).

6 $${\Omega } \approx \pi \left( \frac{a}{d} \right)^{2}$$

where the radius of the detector, *a*, is equal to 2.54 cm, and it was calculated that, in the limit of $d \ge 8a$, the difference between Eqs. (5) and (6) will be negligible. In other words, in the limit of $d \ge 20$ cm, the point source and the NaI(Tl) cylindrical detector can be assumed as a simple two points. Therefore, subtended solid-angle between them can be employed by inverse-square law as (6). So, the ratio of subtended solid-angles (Γ) in the case of presence and absence of height difference can be expressed by (7).

7 $${\Gamma } = \frac{{\Omega }}{{{\Omega }_{{\text{h}}} }} = \frac{{\pi \left( \frac{a}{d} \right)^{2} }}{{\pi \left( {\frac{a}{{d_{{\text{h}}} }}} \right)^{2} }} = \left( {\frac{{d_{{\text{h}}} }}{d}} \right)^{2} = \left( {\frac{{\sqrt {h^{2} + d^{2} } }}{d}} \right)^{2} = 1 + \left( \frac{h}{d} \right)^{2}$$

where Ω_h_ is subtended solid-angle in the presence of the relative height difference between the orphan gamma-ray source and the central axis of the detector. It is obvious that, in the case of large distances (e.g., $d \ge 5$ m), Γ will be very close to unity, and there is no severe difference between Ω and Ω_h_. In the case of smaller distances (e.g., $d \le 5$ m), and by considering that how great h would be, there may be a severe difference between Ω and Ω_h_.

In order to achieve an inductive conclusion, S_1_ and S_2_ ($$E_{1} > E_{2} )$$ are considered in $$\overrightarrow {{P_{1} }} = \left( {d, 0, 0} \right)$$ and $$\overrightarrow {{P_{2} }} = \left( {d, 0, h} \right)$$, respectively. In this case, the distances of S_1_ and S_2_ from detector would be $$d$$ and $$\sqrt {d^{2} + h^{2} }$$, respectively. Assume that, the maximum relative height difference between S_2_ (which has lower energy than S_1_) and the central axis of the detector will not exceed 3 m. By this assumption, Eqs. (5) and (7) can be written as Eqs. (8) and (9), respectively:

8 $$\frac{{d_{{\text{h}}} }}{d} \approx \sqrt {1 + \left( \frac{3}{d} \right)^{2} }$$

9 $${\Gamma } \approx 1 + \left( \frac{3}{d} \right)^{2}$$

Now, the effect of the relative height difference is dependent on the horizontal distance, *d*.

**Figure 6:**
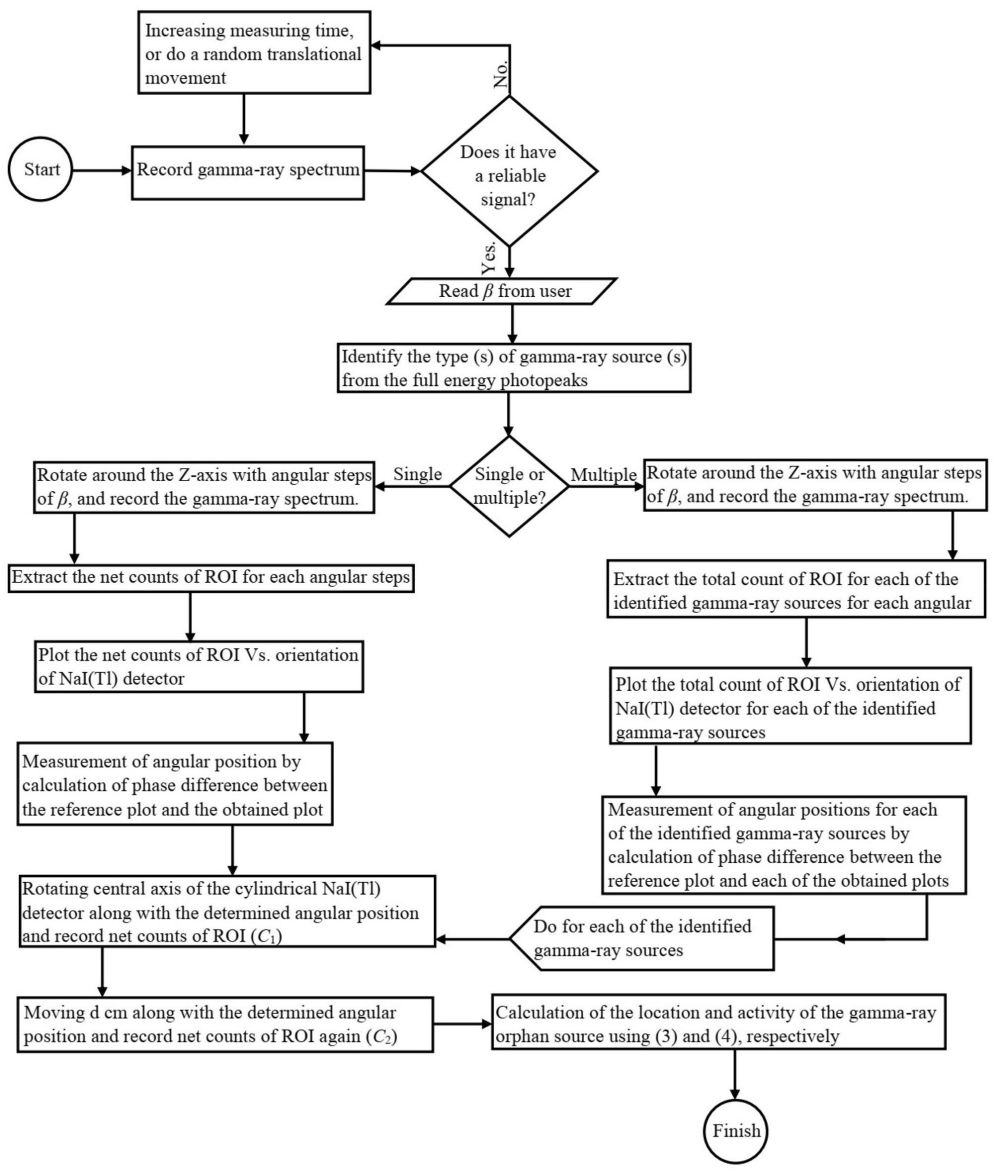
Flowchart of the procedure of finding orphan gamma-ray sources.

To help better understanding of principle of the gamma finder system, whole procedure of steps are illustrated as a flowchart in Fig. 6.

## Results and discussion

In this section, an attempt has been made to evaluate precision and accuracy of the proposed methods in various conditions. As mentioned in the Sect. 2, there are some important factors to characterize the system; (I) relative distances, (II) relative activities, (III) relative height differences of orphan gamma-ray sources. Factors I and II are lying under the fact that the contribution of Compton continuum count due to orphan gamma-ray sources with higher energy is certainly unavoidable. Here, the pairwise ratio of activities as well as distances for gamma-ray sources will be determined. In addition, the third factor; relative height difference; may lead to serious restrictions. In the following, effects of each factor are investigated.

### Relative distances

At first, in the general scenario, there is no information about the positions of the orphan gamma-ray sources. In this stage, it is assumed that two different types of orphan gamma-ray sources are lost in the same height, for example position of the first one (S_1_) is $\vec{r}_{1} = \left( {x_{1} , y_{1} , z_{1} } \right)$ and the second one (S_2_) is $\vec{r}_{2} = \left( {x_{2} , y_{2} , z_{1} } \right)$, but the height of the gamma finder system from the floor, is equal to $z_{0}$, ($z_{1} \ne z_{0}$). It is clear that, this is a simple situation, but the aim of this assumption is to characterize the features of the system and the accuracy of the proposed method. Therefore, in order to achieve this goal, the following procedure is performed:

(1) Consider there are two different types of isotropic orphan gamma-ray point sources with equal activities (by considering their branching ratios), namely, a higher energy source S1 (for example, ^54^Mn), and a lower energy source S2 (for example, ^137^Cs). In general, consider that S1 > S2.

(2) Their gamma spectrum is recorded separately (individually) at certain distance from the NaI(Tl) detector (for example, 5.0 m) and net count of ROI is calculated.

(3) By placing them (individually and not simultaneously) in the certain distance from the detector, the absolute efficiencies of S1 and S2 will be calculated.

(4) As explained before, the MDA of the gamma finder system can be evaluated by (4). In this situation, *N*_B_ is the net count of background in ROI of S_2_. In fact, the net count of S_1_ in ROI of S_2_ is assumed as *N*_B_. If the MDA of the system for S_2_ is lower than the activity of S_1_, then S_1_ goes one step closer toward the NaI(Tl) detector, and MDA of the system for S_2_ is calculated again. This process continues until the calculated the MDA of the system for lower energy orphan gamma-ray source is approximately equal to the activity of higher energy orphan gamma-ray source (^54^Mn). This approach should be repeated for each pair of orphan gamma-ray sources. The results of this approach for orphan gamma-ray sources (Table 1) are presented in Tables 3 and 4.

**Table 3:** Lower limit of simultaneously identified relative distances of gamma sources by considering equal activities (considering branching ratios) from gamma finder (lower energy source is fixed in distance of 2.0 m). “∀” means no matter where S_1_ (*E*_1_ > *E*_2_) is located, if S_2_ is located in distance of 2.0 m from detector, it will be recognizable and “†” indicates that S_2_ will not be identifiable, if S_1_ is located in distance less than 2.0 m from detector.

	^57^Co	^203^Hg	^113^Sn	^103^Ru	^133^I	^137^Cs	^95^Nb	^54^Mn	^60^Co	^40^ K	^24^Na
^57^Co	-	-	-	-	-	-	-	-	-	-	-
^203^Hg	∀	-	-	-	-	-	-	-	-	-	-
^113^Sn	∀	∀	-	-	-	-	-	-	-	-	-
^103^Ru	∀	∀	∀	-	-	-	-	-	-	-	-
^133^I	∀	∀	∀	20.0	-	-	-	-	-	-	-
^137^Cs	∀	∀	7.5	3.5	∀	-	-	-	-	-	-
^95^Nb	∀	∀	7.5	7.0	8.0	∀	-	-	-	-	-
^54^Mn	∀	∀	7.0	6.5	6.0	7.0	13.5	-	-	-	-
^60^Co	∀	∀	8.0	6.5	8.5	13.5	15.5	18.0	-	-	-
^40^ K	∀	∀	∀	∀	∀	∀	∀	∀	∀	-	-
^24^Na	∀	∀	∀	∀	∀	8.5	10.0	13.0	17.5	†	-

**Table 4:** Lower limit of simultaneously identified relative distances of gamma sources by considering equal activities (considering branching ratios) from gamma finder (lower energy source is fixed in distance of 4.0 m). “∀” means no matter where S_1_ (*E*_1_ > *E*_2_) is located, if S_2_ is located in distance of 4.0 m from detector, it will be recognizable and “†” indicates that S_2_ will not be identifiable, if S_1_ is located in distance less than 4.0 m from detector.

	^57^Co	^203^Hg	^113^Sn	^103^Ru	^133^I	^137^Cs	^95^Nb	^54^Mn	^60^Co	^40^ K	^24^Na
^57^Co	-	-	-	-	-	-	-	-	-	-	-
^203^Hg	11.5	-	-	-	-	-	-	-	-	-	-
^113^Sn	8.0	11.5	-	-	-	-	-	-	-	-	-
^103^Ru	12.5	24.5	21.5	-	-	-	-	-	-	-	-
^133^I	12.5	24.0	24.5	91.0	-	-	-	-	-	-	-
^137^Cs	9.0	23.0	29.5	28.5	19.0	-	-	-	-	-	-
^95^Nb	8.5	22.5	41.0	42.5	48.5	23.5	-	-	-	-	-
^54^Mn	8.0	18.0	36.5	38.5	43.5	46.5	59.0	-	-	-	-
^60^Co	8.5	23.5	41.0	42.5	46.0	69.5	78.0	91.0	-	-	-
^40^ K	∀	∀	4.5	4.0	7.5	10.0	12.0	13.5	28.0	-	-
^24^Na	6.0	14.5	31.5	32.5	34.0	49.0	53.0	64.0	80.0	†	-

As mentioned in the section of 2.4, the MDA of the gamma finder system is assumed dependent only on the magnitude of distance ($\left| {\vec{r}} \right|$), and Tables 3 and 4 confirm this claim. The irregularity, as seen in Tables 3 and 4, is because of considering both the energy and the branching ratio of all sources. The reported numbers in Table 3 (Table 4) show that, if S_2_ is located in the distance of 2.0 m (4.0 m) from the detector, then if S_1_ is located closer to the detector, the gamma finder system cannot recognize S_2_. For example, if two isotropic gamma-ray point sources, ^54^Mn and ^133^I, are lost in an environment, and the distance of ^54^Mn from the detector be less than 6.0 cm, then ^133^I will become unrecognizable. As mentioned in the 2.6 section, the point source approximation is valid for $d \ge 20$ cm, so if the reported results in Tables 3 and 4 are smaller than 20 cm, these would not impose any restriction for the system (because it is not within the working aim and scope of the system).

In Tables 3 and 4, the symbol “∀” means no matter where S_1_ (*E*_1_ > *E*_2_) is located, S_2_ will be recognizable. Also, the symbol “†” in Table 3 (Table 4) indicates that S_2_ will not be identifiable, if S_1_ is located in the distance less than 2.0 m (4.0 m) from the detector. As seen in Table 3 all the reported distances are smaller than 20.0 cm. It means that if S_2_ is located in the distance of 2.0 m from detector, no matter what the distance of S_1_ would be and both of them will be recognizable by the gamma finder system (by considering equal activities).

### Relative activities

Suppose two isotropic point gamma-ray sources, namely S_1_ and S_2_, with energies of *E*_1_ and *E*_2_ (*E*_1_ > *E*_2_), respectively, are lost in an environment. Assuming that both of them are placed in the same position. If activity of S_1_ is much greater than, then the system cannot recognize S_2_. The reason is that, photopeak area of S_2_ (or namely, MDA of it) will lie under the Compton continuum of S_1_.

To investigate the effects of relative activities, there is an approach similar to the approach of relative distances. Two isotropic point sources, S_1_ and S_2_, are placed in similar a positions, and the activity of S_1_ will increase gradually until the MDA of the system for detection of S_2_ is equal to the activity of S_1_. The continuation of this investigation is similar to the previous approach, and the results of this investigation are reported in Tables 5 and 6. Dependency of MDA on the magnitude of distance ($\left| {\vec{r}} \right|$) is clearly shown in Tables 5 and 6. It is worth mentioning that, as the designed system can move in the environment (as it is a portable and using an aerial drone), searching for orphan gamma-ray sources in farther distance cannot be a serious restriction.

**Table 5:** Upper limit of simultaneously identified relative activities of gamma sources in distance of 1.0 m from center of gamma finder.

	^57^Co	^203^Hg	^113^Sn	^103^Ru	^133^I	^137^Cs	^95^Nb	^54^Mn	^60^Co	^40^ K	^24^Na
^57^Co	-	99.1	116.3	121.4	105.3	118.8	118.7	123.6	114.2	466.6	139.2
^203^Hg	-	-	96.7	52.9	54.7	60.9	60.2	62.8	56.0	236.7	67.6
^113^Sn	-	-	-	60.0	52.5	33.7	34.0	36.1	33.9	144.4	43.2
^103^Ru	-	-	-	-	16.7	47.7	32.3	35.2	34.0	147.3	43.0
^133^I	-	-	-	-	-	64.1	30.1	32.3	31.8	140.0	40.9
^137^Cs	-	-	-	-	-	-	51.6	31.3	21.5	96.8	28.8
^95^Nb	-	-	-	-	-	-	-	24.7	20.0	92.6	27.2
^54^Mn	-	-	-	-	-	-	-	-	16.8	81.0	23.4
^60^Co	-	-	-	-	-	-	-	-	-	36.9	10.7
^40^ K	-	-	-	-	-	-	-	-	-	-	1.30
^24^Na	-	-	-	-	-	-	-	-	-	-	-

**Table 6:** Upper limit of simultaneously identified relative activities of gamma sources in distance of 2.0 m from center of gamma finder.

	^57^Co	^203^Hg	^113^Sn	^103^Ru	^133^I	^137^Cs	^95^Nb	^54^Mn	^60^Co	^40^ K	^24^Na
^57^Co	-	45.3	55.8	49.7	50.1	54.9	55.5	57.9	52.4	197.2	64.5
^203^Hg	-	-	45.5	25.9	26.5	29.3	29.3	30.6	27.5	104.7	31.9
^113^Sn	-	-	-	28.0	25.3	16.580	16.7	17.5	16.6	65.2	20.4
^103^Ru	-	-	-	-	8.4	23.2	16.0	17.6	16.7	66.7	21.4
^133^I	-	-	-	-	-	30.2	15.1	16.1	15.9	63.9	20.3
^137^Cs	-	-	-	-	-	-	24.6	15.6	10.6	45.1	14.5
^95^Nb	-	-	-	-	-	-	-	12.238	10.2	43.6	13.5
^54^Mn	-	-	-	-	-	-	-	-	8.5	38.4	11.7
^60^Co	-	-	-	-	-	-	-	-	-	15.5	6.1
^40^ K	-	-	-	-	-	-	-	-	-	-	0.6
^24^Na	-	-	-	-	-	-	-	-	-	-	-

### Relative height difference

Based on a mathematical expression which derived to study the effects of the relative height difference in the Materials and Methods section, the mentioned effects are totally is a function of horizontal distance, as described by (9). In Table 7, simple calculations for different values of *d* is presented. Nevertheless, it is difficult and complicated to propose a quantitative expression to clarify the effect of relative height difference between the orphan gamma-ray source and the central axis of the detector. But it can be concluded that, the larger the ratio in Table 7, the higher values reported in Tables 3 and 4, and the lower values in Tables 5 and 6 will be.

**Table 7:** Calculated values of *d*/*d*_h_ and Γ for different values of *d*.

*d* (m)	*d*/*d*_h_	Γ
0.50	6.0	37.0
0.75	4.1	17.0
1.00	3.1	10.0
1.50	2.2	5.0
2.00	1.8	3.2
3.00	1.4	2.0
4.00	1.2	1.5
5.00	1.1	1.3
7.50	~ 1.0	1.1
10.00	~ 1.0	1.1

### Evaluation of the gamma finder system in a hypothetical accident

**Figure 7:**
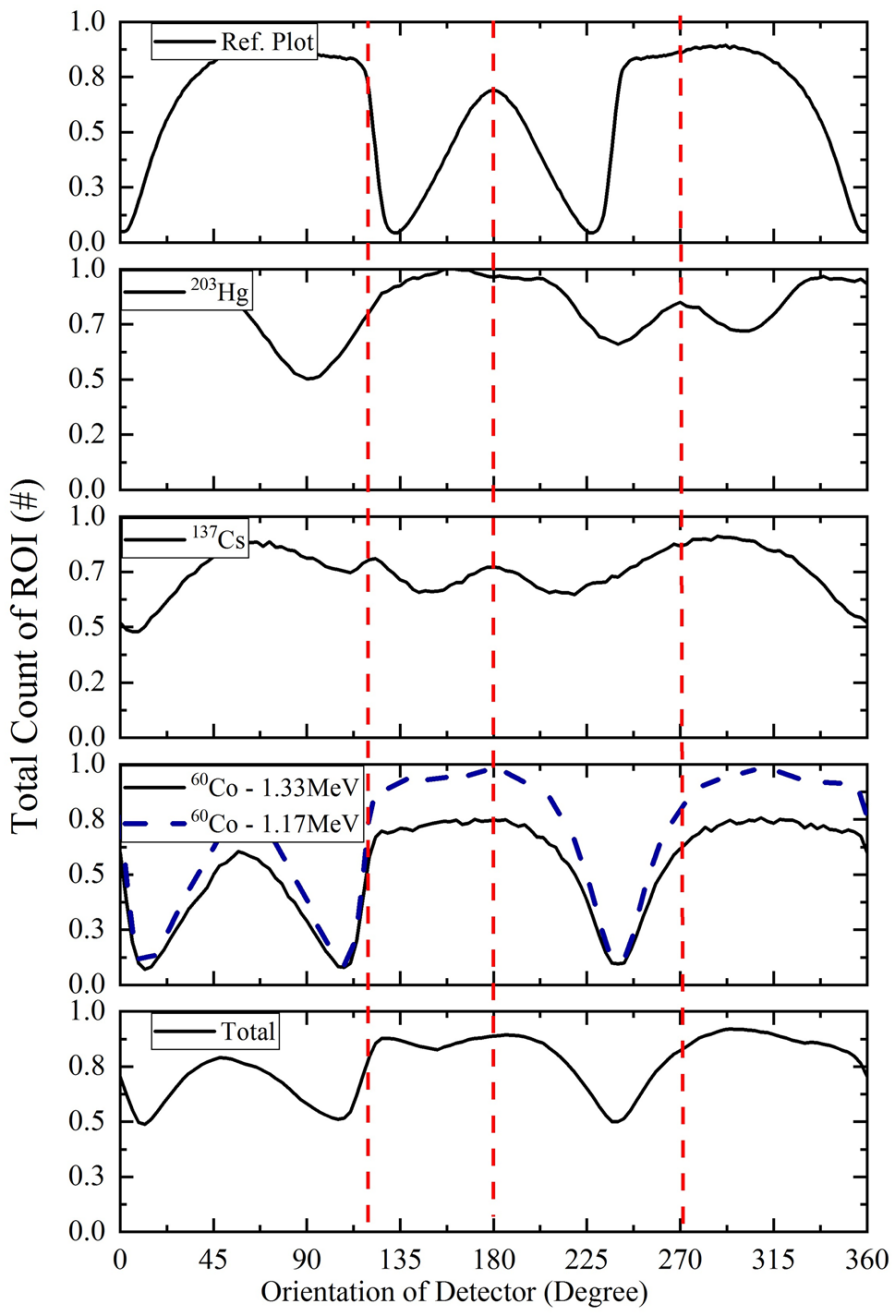
Total count of ROI (per particle) and total count versus orientation of detector in simultaneously presence of ^203^Hg (5 μCi), ^137^Cs (14 μCi) and ^60^Co (25 μCi) in positions of $\overrightarrow {{r_{1} }} = \left( {100, 0, - 35} \right)$ cm, $\overrightarrow {{r_{2} }} = \left( {100\cos \left( {\frac{2\pi }{3}} \right), 100\sin \left( {\frac{2\pi }{3}} \right), 0} \right)$ cm and $\overrightarrow {{r_{3} }} = \left( {100\cos \left( {\frac{3\pi }{2}} \right), 100\sin \left( {\frac{3\pi }{2}} \right),35} \right)$ cm respectively. Each plot in the stack is normalized to the highest value of the total count of ROI of itself.

Here, by considering a hypothetical accident, evaluation of the system will be investigated. In this accident, three orphan isotropic gamma-ray sources have been lost: (1) ^137^Cs with the activity of 14 µCi in $\overrightarrow {{r_{1} }} =$(100, 0, -35) cm, (2) ^60^Co with the activity of 25 µCi in $\overrightarrow {{r_{2} }} =$ (100 cos(π/3), 100 sin(π/3), 0) cm, and (3) ^203^Hg with the activity of 5 µCi in $\overrightarrow {{r_{3} }} =$ (0, 100 cos(π), 35) cm, so the angular positions of ^137^Cs, ^60^Co, and ^203^Hg are 0°_,_ 120°, and 270°, respectively. Initial orientation of central axis of the NaI(Tl) detector in the gamma finder system is considered to be the origin of the angular position. The plot of the total count of ROI versus orientation of the detector for ^137^Cs, ^60^Co, ^203^Hg, and total count (due to presence of three gamma sources simultaneously) is shown in Fig. 7. Based on the reference plot (Fig. 5.a), and measuring the phase difference between each of Fig. 7 and the reference plot, the angular position of each source will be determined. From the plots of Fig. 7, it turns out that ^137^Cs, ^60^Co, and ^203^Hg are placed in 0°, 120°, and 270°, respectively. As mentioned in the Materials and Methods section, it is better to locate and eliminate the higher energy source first, which here it is ^60^Co and thereupon, ^137^Cs. As reported in Table 8, the relative error in determining position and activity of the mentioned orphan gamma-ray sources in this fictitious accident is approximately less than 5% and 8%, respectively (by considering the elimination of higher energy sources, one by one, e.g., first ^60^Co, and thereupon ^137^Cs). As mentioned above, it is recommended that, after recognizing all the orphan gamma-ray sources, first determine the position and the activity of source with the highest energy among all of them, and eliminate it, thereupon go for the next ones. It should be considered that, when the angular position of each of them is determined, the central axis of the NaI(Tl) detector will be rotated along the each of the determined the angular position. Therefore, hence the position of each orphan gamma-ray source in two dimensions, will be reduced to one dimension which is called the horizontal distance.

**Table 8:** Relative error in determining positions and activities of ^60^Co, ^137^Cs and ^203^Hg in the hypothetical accident.

	^60^Co	^137^Cs	^203^Hg
Exact horizontal distance (cm)	100	100	100
Calculated horizontal distance (cm)	95.5	97	98
Relative error in calculation of horizontal distance	4.5	3	2
Exact activity (μCi)	25	14	5
Calculated activity (μCi)	23.2	12.8	4.7
Relative error in calculation of activity (%)	7.0	8.0	5.0

**Figure 8:**
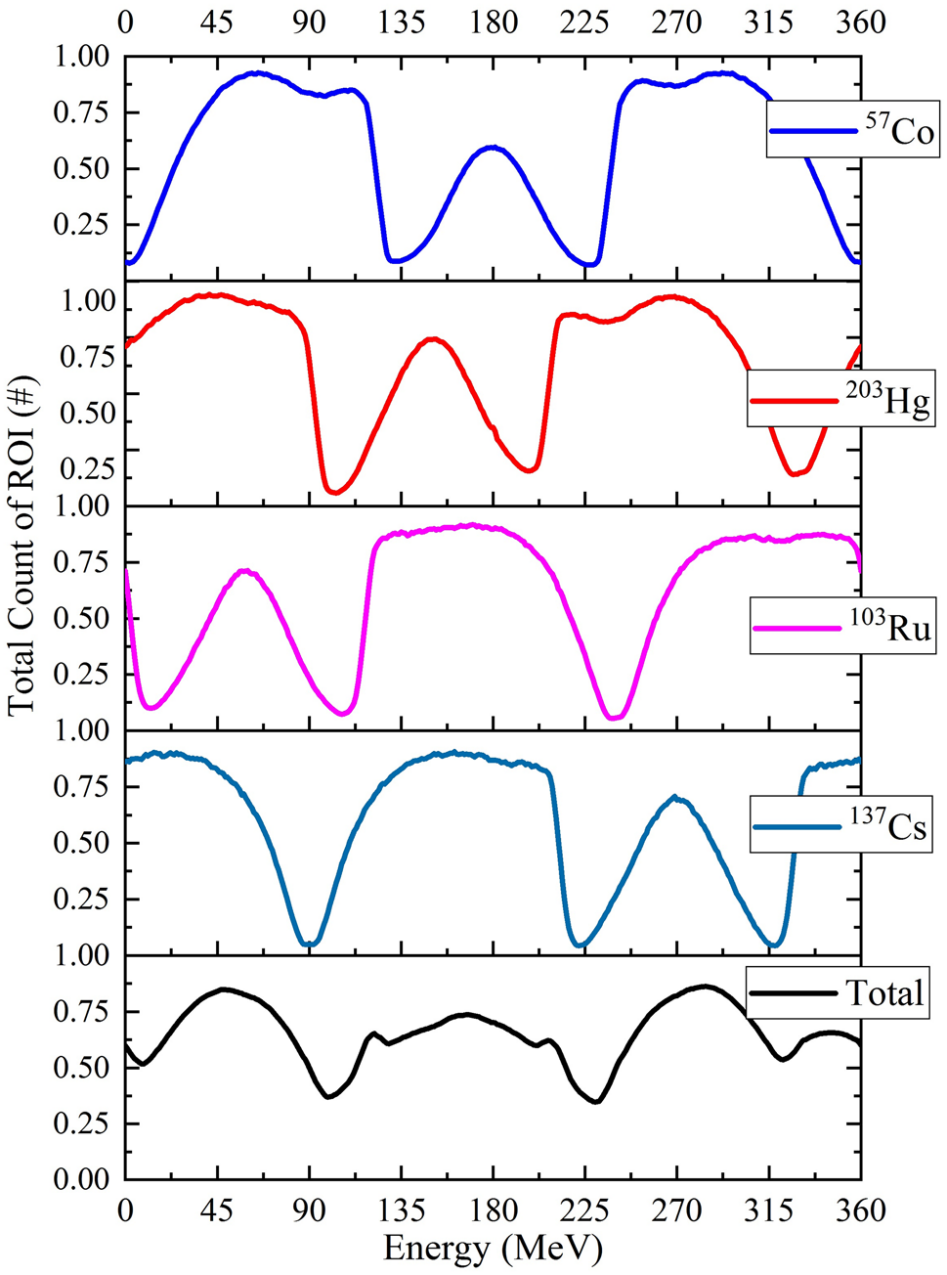
Total count of ROI (per particle) and total count versus orientation of detector in simultaneously presence of ^57^Co, ^203^Hg, ^103^Ru and ^137^Cs in positions of $\vec{r}_{1} ec = \left( {100, 0, 0} \right)$ cm, $\vec{r}_{2} = \left( {100\cos \left( {\frac{\pi }{6}} \right), 100\sin \left( {\frac{\pi }{6}} \right), 0} \right)$ cm and $\vec{r}_{3} = \left( {100\cos \left( {\frac{2\pi }{3}} \right), 100\sin \left( {\frac{2\pi }{3}} \right),0} \right)$ cm and $\vec{r}_{4} = \left( {100\cos \left( {\frac{3\pi }{2}} \right), 100\sin \left( {\frac{3\pi }{2}} \right), 0} \right)$ respectively. Each plot in the stack is normalized to the highest value of the total count of ROI of itself.

Here, another hypothetical accident was simulated and presented. In this accident, there are 4 orphan gamma-ray sources (with equal activities), ^57^Co in $\vec{r}_{1} = \left( {100, 0, 0} \right)$, ^203^Hg in $\vec{r}_{2} = \left( {100\cos \left( {\frac{\pi }{6}} \right), 100\sin \left( {\frac{\pi }{6}} \right), 0} \right)$, ^103^Ru in $\vec{r}_{3} = \left( {100\cos \left( {\frac{2\pi }{3}} \right), 100\sin \left( {\frac{2\pi }{3}} \right), 0} \right)$, and ^137^Cs in $\vec{r}_{4} = \left( {100\cos \left( {\frac{3\pi }{2}} \right), 100\sin \left( {\frac{3\pi }{2}} \right), 0} \right)$. So according to the gamma-ray sources’ positions, it is obviously known that the angular position of ^57^Co, ^203^Hg, ^103^Ru and ^137^Cs are 0^0^, 30^0^, 120° and 270°, respectively (see Fig. 8). Based on the reference plot (Fig. 5a), and measuring the phase difference between each of the above figure and the reference plot, the angular position of each source will be determined. From the plots of above figure, it turns out that ^137^Cs, ^103^Ru, ^203^Hg and ^57^Co are placed in 270°, 120°, 30^0^ and 0°, respectively. It is worth mentioning that, no useful information can be deduced from the curve of the total counts in the above figure (the bottom curve). It is obvious that, for more number of orphan gamma-ray sources in an accident, a similar approach will be taken.

As far as we know based on the available reported researches, the current designed systems cannot find multiple orphan gamma-ray sources (even two gamma-ray sources)^5–15^. It is worth mentioning that a Pb-collimator is implemented in some of the current designed systems, which able to find multiple orphan gamma-rays, but when the gamma-ray orphan sources are placed in a significant height out of view of collimator, for example 1.0 m or 2.0 m above the collimator, the system cannot find the orphan gamma. The advantages and disadvantages of the gamma finder system are listed in Table 9.

**Table 9:** The advantages and disadvantages of the gamma finder system.

Advantages	Able to work properly in single/multiple orphan gamma-ray sources scenario
	Able to work properly when orphan gamma-ray sources are placed in different heights
	Affordable, as it uses only one real NaI(Tl) detector
	Simple electronics equipment and approach, as it uses only one real NaI(Tl) detector
	Able to work for wide range of gamma-ray energies
	Able to work in high background gamma-ray radiation from other gamma-ray sources
Disadvantages	Mass about 14 kg which will be tried in future studies to reduce it
	Not able to find position of two orphan gamma-ray sources with similar energy.

Overall, this system has a notable imperfection; if there are two or more similar lost gamma-ray sources (in various positions) in an environment, the system can find none of them. It is because of that, all of them will contribute in the total count of ROI and therefore a reasonable curve of total count of ROI versus orientation of detector cannot be obtained. Although there is no report about multiple sources scenario in the database of radiological events and related events^1^, but it can be a possible accident. For example, suppose an accident which, a gamma-ray source is lost in a laboratory and there are gamma-ray sources which are using in experiments. So, in this case, the gamma finder system must be able to find the gamma-ray orphan source in presence of significant gamma-ray background radiation (due to the other gamma-ray sources in the laboratory). The designed system should work without any fatal error in various conditions, especially in presence of multiple gamma-ray sources. In other words, an ideal and perfect system should be designed in such a way that all aspects and conditions of accidents are considered with related solutions.

## Conclusion

In this research, development of gamma finder system in 3D localization for multiple gamma sources scenario is presented. A real Ø2″ × 2″ cylindrical NaI(Tl) scintillation detector and two fake detectors are mounted on an aerial drone. All the movement, elevation and rotation (around the Z-axis) of the system is done by drone. Now, the developed methods have enabled this system to work correctly and precisely in multiple sources scenario. Based on the results, this system may have some considerations and restrictions in special cases that discussed in detail. But in general and most cases, this system can find multiple orphan gamma-ray sources in various situations and conditions. In general, in a reasonable condition which relative distances and relative activities of orphan gamma-ray sources are not too far from each other, the proposed system can find all the listed gamma-ray sources in Table 1 simultaneously. The total mass of the gamma finder system is evaluated in Sect. 2 as less than 14 kg, and some commercial aerial drones can be used for the current gamma finder system, but in the next development program, some efforts will be made to reduce the total mass. As mentioned in the Results and discussion section, however there are no report about multiple similar orphan gamma-ray sources, but if such an accident happens, the proposed methods in this research will not be effective in finding them. However, providing an approach to solve the mentioned problem is not seeming easy here, but may be possible to determine angular position of them by using partial scanning of horizontal field of view, or by employing another real radiation detector. Anyway, a part of the further studies, will focus on solving the mentioned problem. In addition to the above, remaining part of further and future studies, may involve testing this system in practical fields.
